# RNA Polymerases IV and V Are Involved in Olive Fruit Development

**DOI:** 10.3390/genes15010001

**Published:** 2023-12-19

**Authors:** Alicia Serrano, Martín Moret, Isabel Fernández-Parras, Aureliano Bombarely, Francisco Luque, Francisco Navarro

**Affiliations:** 1 Instituto Universitario de Investigación en Olivar y Aceites de Oliva, Universidad de Jaén, 23071 Jaén, Spain; asg00144@ext.ujaen.es (A.S.); mmoret@ujaen.es (M.M.); ifparras@ujaen.es (I.F.-P.); 2 Instituto de Biología Molecular y Celular de Plantas (IBMCP), CSIC and Universitat Politécnica de Valencia, 46011 Valencia, Spain; abombarely@ibmcp.upv.es; 3 Departamento de Biología Experimental, Universidad de Jaén, 23071 Jaén, Spain

**Keywords:** RNA polymerases, long non-coding RNAs, olive, stress conditions, fruit development

## Abstract

Transcription is carried out in most eukaryotes by three multimeric complexes (RNA polymerases I, II and III). However, plants contain two additional RNA polymerases (IV and V), which have evolved from RNA polymerase II. RNA polymerases II, IV and V contain both common and specific subunits that may specialise some of their functions. In this study, we conducted a search for the genes that putatively code for the specific subunits of RNA polymerases IV and V, as well as those corresponding to RNA polymerase II in olive trees. Based on the homology with the genes of *Arabidopsis thaliana*, we identified 13 genes that putatively code for the specific subunits of polymerases IV and V, and 16 genes that code for the corresponding specific subunits of polymerase II in olives. The transcriptomic analysis by RNA-Seq revealed that the expression of the RNA polymerases IV and V genes was induced during the initial stages of fruit development. Given that RNA polymerases IV and V are involved in the transcription of long non-coding RNAs, we investigated their expression and observed relevant changes in the expression of this type of RNAs. Particularly, the expression of the intergenic and intronic long non-coding RNAs tended to increase in the early steps of fruit development, suggesting their potential role in this process. The positive correlation between the expression of RNA polymerases IV and V subunits and the expression of non-coding RNAs supports the hypothesis that RNA polymerases IV and V may play a role in fruit development through the synthesis of this type of RNAs.

## 1. Introduction

Transcription in bacteria and archaea is carried out by a single multimeric RNA polymerase, while most eukaryotes contain three multimeric complexes (RNA pol I, II, and III) [1,2,3]. Furthermore, plants contain two additional RNA pols (IV and V) that have evolved from RNA pol II [4,5,6,7,8,9,10,11,12]. RNA pol I consists of 14 subunits and synthesises precursor rRNA 45S (35S in yeast) of the three largest rRNAs [3,13,14,15]. RNA pol III contains 17 subunits and transcribes tRNAs, 5S rRNA and other non-coding RNAs [13,16,17,18]. RNA pol II is composed of 12 subunits and synthesises mRNAs and some non-coding RNAs [13,19,20,21]. Plant-specific RNA pol IV and V, which have evolved from RNA pol II through duplication and functional divergence, also contain 12 subunits. These two enzymes that are involved in epigenetic regulation to synthesise siRNAs play roles in transcriptional silencing via RNA-directed DNA methylation (RdDM), and in non-coding RNAs’ participation in plant growth, development, response to environmental changes or plant immunity [4,5,6,7,8,9,10,11,12,22,23,24]. 

RNA pol II, IV and V contain specific conserved subunits that may specialise some of their functions [5,22,25]. This is the case of subunits NRPD1, NRPE1 and NRPB1 (nuclear RNA polymerase subunit 1) which correspond to RNA pols IV, V and II, respectively, in *A. thaliana*. The specific functions of NRPD1 and NRPE1 highlight the specialisation of these RNA polymerases in mediating distinct aspects of the RdDM process, while NRPB1 associates with mRNA and certain ncRNA synthesis [5,22,25]. In addition, there are other subunits that are common to RNA pol IV and V, but are conserved in RNA pol II, such as the subunits NRPDE2 and NRPB2 (nuclear RNA polymerase subunit 2), RNPDE4 and NRPB4 (nuclear RNA polymerase subunit 4), and NRPDE7 and NRPB7 (nuclear RNA polymerase subunit 7), which are shared by RNA pol IV and V and conserved with RNA pol II [5,22,25,26,27]. Furthermore, several isoforms of the common subunit five, shared by all the RNA pols have been described, while a specific isoform, NRPE5 (nuclear RNA polymerase subunit 5), has been found for RNA pol V [28]. In addition, several paralogues have been described for these and other subunits in different plants [22,25,26,28,29,30]. Based on the existence of these paralogues, it has been proposed that these may perform new functions or be subject to different regulation. This is indeed the case of the distinct isoforms of the shared subunits from the RNA pols in cultivated olive trees “Picual” (*Olea europaea* L. cv. Picual) [28].

RNA pol IV and V have been reported to be involved in the biogenesis and functionality of 24 nt siRNA, which participates in RdDM [9,12,25,30]. RNA pol IV and V have also been proposed as participating in the transcription of long non-coding RNAs (lncRNAs) [24,31,32,33,34,35,36,37,38]. Some of these lncRNAs are the intermediary of siRNA and are found within intergenic regions [34]. Although lncRNAs are also transcribed by RNA pol II, those synthesised by RNA pol IV and V show some structural differences as regards the RNA pol II ones, such as lack of poly-A at the 3′ end region or lack of introns [4]. lncRNAs transcribed by RNA pol IV and V are poorly characterised, in part because of their low expression and instability [34,39]. However, well-studied examples of non-polyA lncRNAs have been reported [40,41,42]. Notably, the synthesis of non-polyA lncRNAs can be regulated by environmental conditions, as demonstrated in *A. thaliana* under abiotic stress [43,44,45].

In this work we searched for genes that putatively encode specific subunits of RNA pol IV and V, and for those corresponding to RNA pol II, in the olive “Picual” cultivar given its economic, agronomic and agro-ecological importance as one of the most important fruit trees in the Mediterranean Basin [28,46,47,48]. The analysis allowed us to identify paralogues for NRPD1, NRPE1 and NRPB1, NRPDE2 and NRPB2, NRPDE4 and NRPB4, NRPDE7 and NRPB7, and also for NRPB7-like, in addition to the putative pseudogenes, according to our transcriptomic analyses. The transcriptional studies from RNA-Seq data evidenced an increase in the corresponding RNA pol IV and V genes during fruit development. Furthermore, given the known role of RNA pol IV and V in the transcription of lncRNAs, we studied the lncRNA transcriptome during fruit development, which revealed significant changes in their expression and differed based on the analysed lncRNA type. Accordingly, our data point to the involvement of RNA pol IV and V in the regulation of lncRNAs during fruit development.

## 2. Materials and Methods

### 2.1. Plant Material

In order to analyse gene expression during fruit development in olive tree, flowers and fruit were collected from the “Picual” olive cultivar growing in the experimental field of the University of Jaén (Jaén, Spain). Flower and fruit samples were collected from three different closely located trees and from south-facing branches to reduce environmental variability, as specified by [49]. Therefore, three independent biological samples were collected at eight different times from full bloom (flowering) to fruit ripening,15 days after full bloom (AFB), and monthly from 1 to 6 months AFB. These samples were immediately frozen in liquid nitrogen and stored at −80 °C for RNA extraction.

Plant samples of the “Picual” cultivar were used to study the response to root injury, *Verticillium dahliae* infection, and cold stress. They were obtained from the Department of Crop Protection, Institute for Sustainable Agriculture, Córdoba, Spain, and described previously [50,51]. Briefly, to study root injury and *V. dahliae* infection, plants were infected by immersion inoculation of roots in a conidial suspension (10^7^ conidia ml^−1^) of defoliant *V. dahliae* isolate V937I. To analyse root injury, plants were manipulated in a similar way but without inoculating the pathogen. To reduce variability between plants, RNAseq samples consisted of three biological replicates, each one obtained from three pooled plant roots taken at 0, 2 and 7 days after infection or injury. The cold stress experiment was carried out with 4-month-old plants, acclimated at 24 °C, and then incubated with a 14 h photoperiod of fluorescent light at 65 μmol m^2^ s (10 °C day/4 °C night) for 10 days and constant relative humidity between 76 and 78%. Aerial tissues were collected at 0, 10, and 24 days, and three biological replicates were obtained in pooled samples.

### 2.2. Transcriptomic Analysis

The total RNA from the triplicate samples of flowers and fruit at 15 days AFB was isolated using the Spectrum™ Plant Total RNA Kit (Sigma-Aldrich, St. Louis, MO, USA) according to the manufacturer’s instructions. PoliA+ RNA was purified and sequenced from the samples collected during fruit development as indicated by [49]. Briefly, poliA+ RNA 150 bp × 2 paired-end Illumina sequences were obtained from Novogene (UK) and at least 50 M reads of Q30 sequences data were obtained from each biological replicate sample. The dataset is available at NCBI as BioProject: PRJNA870905. 

For this work, an additional RNA-Seq of the total RNA was carried out. In this case, 150 bp × 2 paired-end Illumina sequences were obtained at Ascires (Valencia, Spain) from the flower and 15-day AFB samples, as well as a mix of RNAs from flower, fruit, root, leaf, meristem and stem. For total RNA sequencing, at least 100 M reads were obtained per sample. The dataset is available at NCBI as BioProject: PRJNA989401.

Other RNA-Seq data were used as described in [28]. Basically, a previous RNA-Seq from olive organ/tissues [52] and several stresses, such as cold, injury or *V. dahliae* infection [51], were analysed. The datasets are available at BioProject PRJNA556567 and at NCBI accession numbers SRR1525051, SRR1525052, SRR1524949, SRR1524950, SRR1524951, SRR1524952, SRR1525086, SRR1525087, SRR1525113, SRR1525114, SRR1525231, SRR1525237, SRR1524947, SRR1524948, SRR1525213, SRR1525114, SRR1525224, SRR1525226, SRR1525284, SRR1525285, SRR1525286, SRR1525287, SRR1525415, SRR1525416, SRR1525436, and SRR1525437.

The RNA-Seq analysis was performed with DNAstar (ArrayStar 17, Rockville, MD, USA) for the RNA-Seq analyses (www.dnastar.com, accessed on 20 November 2022). Gene expression was carried out using a 95% false discovery rate (FDR).

### 2.3. Annotation of lncRNAs in Olive

Assessments of raw sequence quality were first performed using the FastQC software (version 0.11.5, http://www.bioinformatics.babraham.ac.uk/projects/fastqc/, accessed on 16 January 2023). Adapter sequences and reads shorter than 50 bp were trimmed with Fastq-mcf (EA-Utils version 1.04.759) (http://expressionanalysis.github.io/ea-utils/, accessed on 18 January 2023). Next, clean reads were mapped to the olive genome of the “Picual” cultivar [53] available in OliveTreeDB (https://genomaolivar.dipujaen.es/db/downloads.php, accessed on 20 January 2023) using the HISAT2 software (v2.2.1) [54]. Mapped reads were sorted and compressed by Samtools (v1.16.1) [55], and then assembled and merged using StringTie v2.2.1 [56]. The gffcompare tool (v0.12.6) [57] was used to identify the unannotated transcripts by comparing the assembled transcriptome to the reference “Picual” transcriptome. Note that these unannotated transcripts corresponded to the non-polyA lncRNAs. Subsequently, the transcripts categorised as “u” (intergenic lncRNAs), “x” (antisense lncRNAs), “i” (intronic lncRNAs) and higher than 200 bp were selected as candidate lncRNAs. 

**Figure 1:**
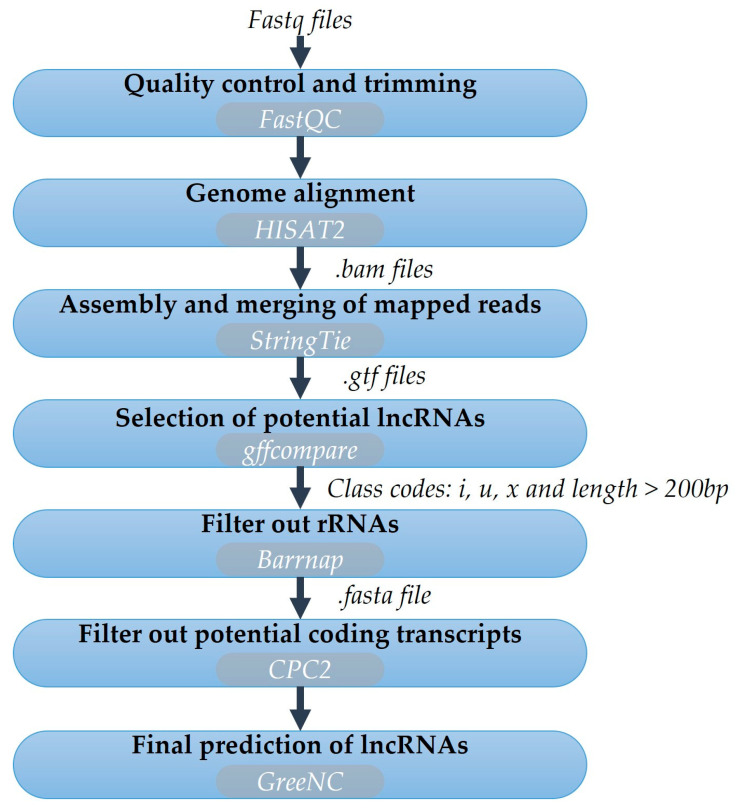
LncRNAs identification pipeline from RNAseq datasets.

However, as the selected transcripts could contain coding genes, they underwent another filtering process. The transfer RNAs were filtered using the tRNAscan-SE 2.0 tool [58]. Barrnap tool v0.7 (https://github.com/tseemann/barrnap, accessed on 13 March 2023) was applied to identify the ribosomal RNA genes and CPC2 software v2.0 (http://cpc2.cbi.pku.edu.cn, accessed on 13 March 2023) [59] was applied to filter out those transcripts with coding ability. Finally, transcripts were analysed by the second script of GreeNC v2.0 (https://github.com/sequentiabiotech/GreeNC, accessed on 16 March 2023) to discriminate any other non-coding transcripts from lncRNAs and to identify any possible miRNA precursors (Figure 1).

### 2.4. Analysis of the Differentially Expressed lncRNAs

The expression analysis was performed with DNAstar (ArrayStar 17, Rockville, MD, USA, www.dnastar.com (accessed on 20 March 2023)). Mapping was carried out with high-stringency parameters to differentiate between very similar paralogues, k-mer = 63 and 95% matches. Data were normalised based on reads per kilobase of transcript per million reads mapped (RPKM). A basal expression level of log2 RPKM = −2 was considered. Therefore, the genes with expression values above this threshold level were considered expressed, whereas those genes with expression values that equalled or were below the threshold level were considered not expressed. A comparison between samples was made using the parametric *t*-student test.

## 3. Results

We conducted a genome search for olive genes that are highly homologous to those that code for the specific subunits of RNA pol IV and V in *A. thaliana*. Then, their expression profile was studied by RNA-Seq in different plant organs/tissues in response to environmental stresses, as well as during the fruit development process. As RNA pol IV and V have evolved from RNA pol II, the equivalent olive genes for the RNA pol II subunits were also identified. These genes coding for the RNA pol II subunits were used as references for the expression profiles and were compared to the RNA pol IV and V coding genes. An additional annotation and expression analysis of lncRNAs was also performed.

### 3.1. Olive Genes Coding for RNA Pol IV and V Subunits

**Figure 2:**
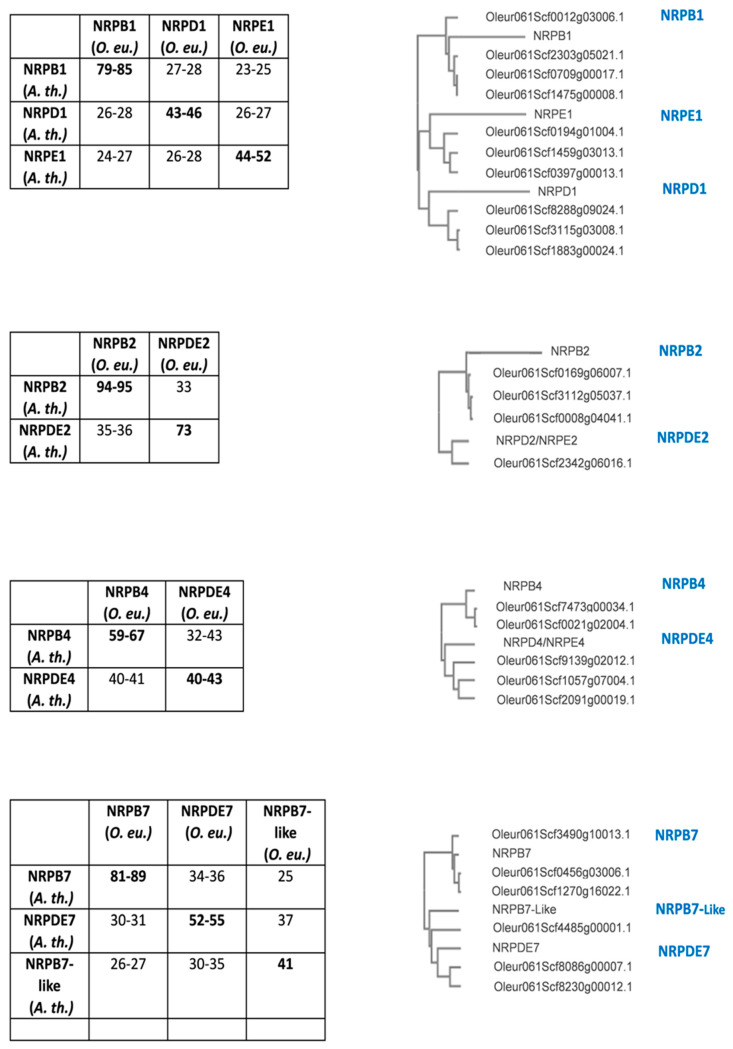
(**Left panel**) Amino acids’ identities calculated with the multialignment tool of Blast-P. (**Right panel**) Cladograms generated with Clustal Omega. The *A. thaliana* subunits are denoted by their NRP name.

In order to search for the genes putatively coding for the specific subunits of RNA pol IV and V in olives, the *A. thaliana* sequences for NRP1, NRP2, NRP4 and NRP7 of RNA pol II, IV and V were used as a query to identify the corresponding homologues. This search yielded several paralogues: 16 for pol II and 12 for RNA pol IV and V (Table 1 and Figure 2). This was not surprising because olive ancestors have quite probably undergone two whole genome duplication (WGD) events in the last 65 M years [53,60]. Furthermore, putative pseudogenes were identified for RNA pol II which were not expressed and contained inactivating mutations according to our RNA-Seq analyses under different conditions (see below).

### 3.2. Gene Expression Profile in Different Plant Organs/Tissues

**Figure 3:**
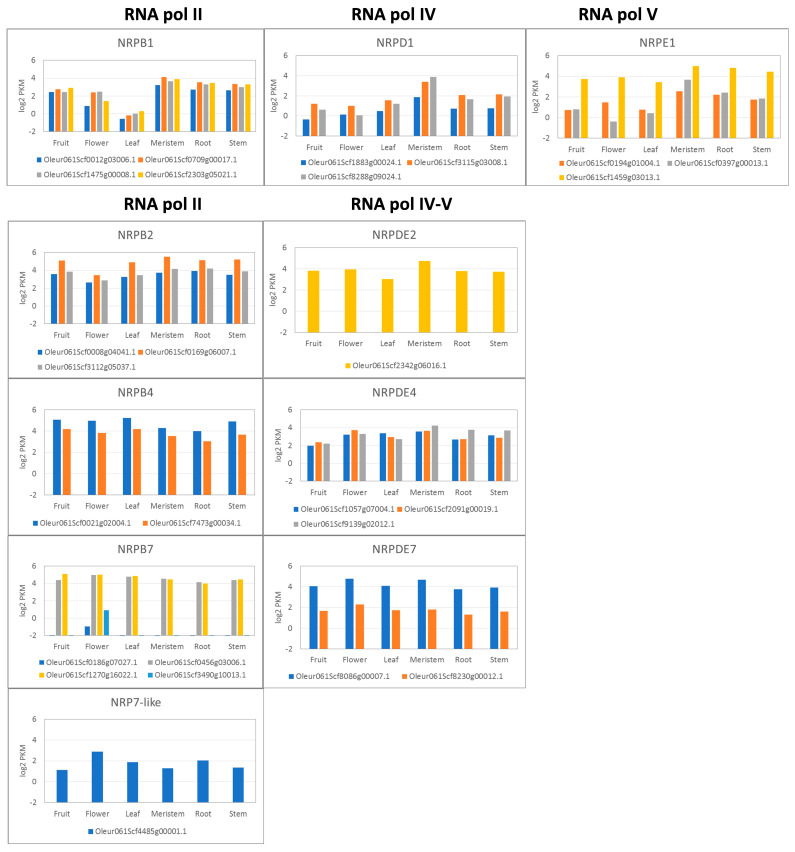
Expression profile of RNA pol II, IV and V subunits in different olive tree organs.

In a previous study, we found that the genes coding for subunits shared by RNA pols in “Picual” cultivar [28] were spatially and temporally regulated. To investigate whether the specific subunits of the RNA pol II, IV and V are spatially regulated in plants, a transcriptomic analysis was performed. The transcriptomic analysis of the genes coding for the specific subunits of RNA pol II, IV and V showed that some of them were regulated in the different analysed plant tissues (Figure 3). In line with this, the genes coding for RNA pol II subunits 1, 2, 4 and 7 generally exhibited a relatively uniform expression across the different analysed tissues, with some exceptions. For instance, the four genes coding for subunit 1 of RNA pol II (NRPB1) showed a lower expression level in leaves than in the other tissues. Similarly, two genes coding for subunit 7 (NRPB7), specifically *Oleur061Scf0186g07027.1* and *Oleur061Scf3490g10013.1*, seemed to be tissue-specific because they were expressed only in flowers. Furthermore, one of the genes coding for subunit 7 of RNA pols IV and V (*Oleur061Scf8086g00007.1*) showed the highest expression level for all tissues. In contrast, the putative pseudogenes of the NRPB7 and NRPB7-like subunits were not expressed in any organ or tissue. Regarding the RNA pol IV and V subunits, the overall expression pattern was also quite homogeneous, except for the NRPD1 and NRPE1 subunits, which seemed to be expressed at variable levels depending on the specific plant tissue.

### 3.3. Expression Profile in Response to Biotic and Abiotic Stresses

RNA pol IV and V might be involved in the response to stress stimuli by plants, according to reported data [12,61]. To examine this possibility, we studied the expression level of those genes coding for the different RNA pol II, IV and V subunits in response to root injury, *V. dahliae* infection and cold stress. As a result of the transcriptomic analysis of the genes encoding the specific RNA pol II, IV and V subunits (Figure S1), no consistent expression pattern in response to any of the studied stresses was observed. However, for root injury stress, some of the genes coding for the RNA pol IV and V subunits seemed to slightly reduce their expression following injury, and the original expression levels were recovered after a 7-day follow-up. This behaviour was not consistent among all the subunits or among all the paralogue genes of the same subunit.

In addition, no specific response to biotic stress produced by *V. dahliae* infection was detected (Figure S2). In this case, minor changes were similar to the response observed in the case of root injury, which was performed to induce *V. dahliae* infection. Notably, some major changes were observed after 15 days post-inoculation. At the time of this follow-up, plants displayed clear severe disease symptoms. This fact could modify the general gene expression pattern, as previously described by [50]. 

Regarding response to cold stress, changes in the expression for some genes of the RNA pol II, IV and V specific subunits were observed, but, once again, no consistent pattern was found (Figure S3).

### 3.4. Expression Profile during Fruit Development

In line with the reported role of RNA pol IV and V in plant development, we investigated whether this could be the case in olive trees. To investigate this, we performed a transcriptomic analysis of the genes putatively coded for the specific RNA polymerases II, IV and V subunits during fruit development. For this purpose, samples from three trees were analysed by RNA-Seq, which consisted of recently bloomed flowers and developing fruit at 15 days and every month from the flowering stage to fully ripe fruit (Figure S4) [49]. 

**Figure 4:**
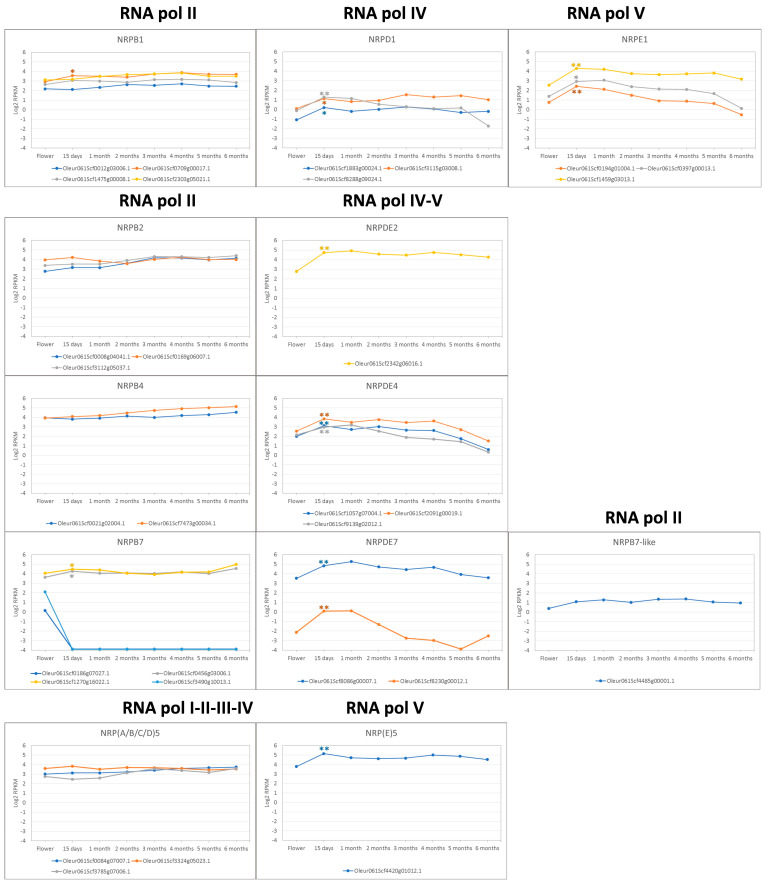
Expression profile of RNA pol II, IV and V subunits during fruit development. * *p*-value < 0.05 and ** *p*-value < 0.01 at comparing flower and after 15 days of fruit development.

Notably, all the RNA pol IV and V specific subunits showed a significant induction at 15 days after full bloom (AFB) (Figure 4), with an average fold change of 3.2 (1.8–8.0). However, this induction at 15 days AFB was observed for only three of the genes of RNA pol II (*Oleur061Scf0709g00017.1* of subunit 1; *Oleur061Scf1270g16022.1* and *Oleur061Scf0456g03006.1* for subunit 7), with a fold change from 1.3 to 1.6. Therefore, no relevant changes in the gene expression were observed for the genes of the specific RNA pol II subunits, except for the NRPB7 genes *Oleur061Scf0186g07027.1* and *Oleur061Scf3490g10013.1* which, according to the organ/tissue specificity proposed above (Figure 2), were rapidly repressed once fruit development began. In addition, the two putative pseudogenes identified for NRPB7 and NRPB7-like were not expressed during fruit development.

Olive cultivar “Picual”, like other plants, contains a gene that codes for the specific RNA pol V subunit NRPE5, an isoform of subunit NRP5 shared by all eukaryotic RNA polymerases [28]. In line with this protein being specific for RNA pol V, the corresponding gene (*Oleur061Scf4420g01012.1*) showed a significant induction on the first 15 days of fruit development (Figure 4). Note that this was not the case in the other NRP5 paralogues identified in olive trees.

Taken together, these data suggest that RNA pol IV and V may play a major role in early fruit development steps. As RNA pol IV and V have been proposed to participate in the transcription of lncRNAs [24,31,32,33,34,35,36,37,38], we can speculate about a transcriptional response by these types of transcripts during this development process.

### 3.5. Annotation and Expression of lncRNAs

**Figure 5:**
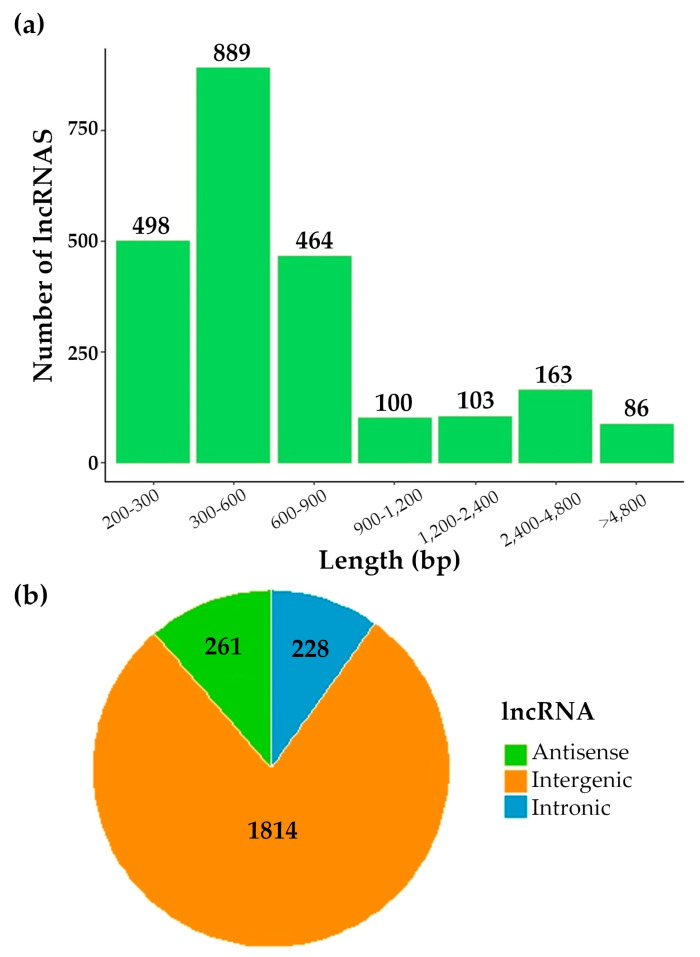
Classification of lncRNAs. (**a**) Length of lncRNAs; (**b**) location of lncRNAs classified based on their genomic location regarding the neighbouring protein-coding genes: intergenic (transcript mapped to the unknown intergenic regions), intronic (transcripts mapped completely within the introns of the known protein-coding genes), and antisense (transcript mapped to the exon of the protein-coding gene but on the opposite strand).

In order to study the expression pattern of lncRNAs in olive trees, six strand-specific RNA-seq libraries were constructed (with three biological replicates each), using the total RNA of the olive flower at full bloom and the olive fruit at 15 days AFB. An additional mix including different plant tissues was sequenced to obtain a broad representation of the lncRNA transcriptome in the olive. From the total reads showing high quality (score > Q30), 744,825,368 clean reads were obtained from the seven libraries after trimming the adapters and reads that were shorter than 50 bp (Table S1). These clean reads were aligned to the olive genome of the “Picual” cultivar (https://genomaolivar.dipujaen.es/db/downloads.php, accessed on 20 November 2022). Alignment rates appeared to range from 60.24% to 86.07% (Table S1). Subsequently, 120,670 total transcripts were assembled using Stringtie, and 79,654 transcripts resulted after filtering the transcripts by size ≥ 200 bp. The length of the lncRNAs ranged from 200 bp to 21,212 bp, although most were shorter than 900 pb (Figure 5a). These transcript sequences were analysed to identify putative lncRNAs. As a result, 3603 total transcripts from both experimental conditions were selected as intergenic, intronic or antisense after annotation with GffCompare. No tRNAs were identified when applying tRNAscan-SE. Furthermore, 146 rRNAs and 370 coding transcripts were discarded by applying Barrnap and CPC2, respectively. Finally, 2303 candidate non-poly-A lncRNAs were identified as non-coding RNAs by GreenNC, including 1814 intergenic, 261 antisense and 228 intronic transcripts (Figure 5b).

**Figure 6:**
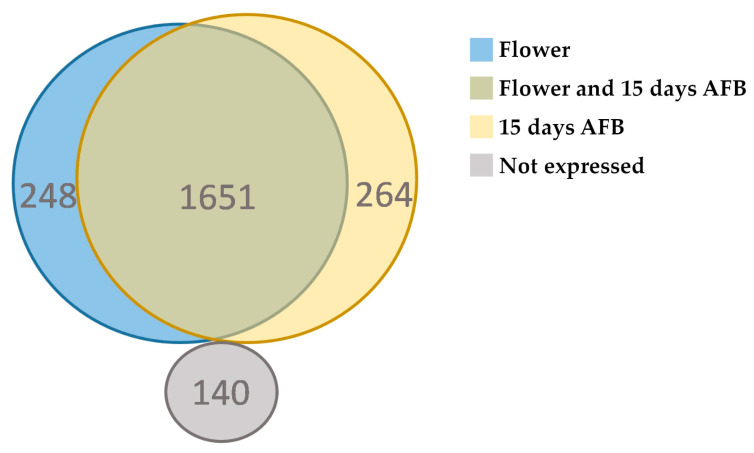
Venn diagram showing the expression pattern in flowers and developing fruits at 15 days AFB.

The analysis allowed us to identify 1899 lncRNAs (non-polyA lncRNAs) in flowers and 1915 from 15 days AFB, with 1651 in common (Figure 6). In addition, changes in the expression pattern of lncRNAs were observed. During the transition from flowering to 15 days AFB, 143 lncRNAs were found to be upregulated and 273 downregulated by using a false discovery rate (FDR) of 5% (Table 2). However, no major changes in the average expression of lncRNAs were found between the flower and 15-day AFB samples, with 718.68 and 733.22 RPKMs, respectively (*p*-value = 0.8365). Nevertheless, some differences between the flower and the 15-day AFB samples were observed when discriminating between types of lncRNAs (Table 2). Specifically, a tendency towards increased expression in lncRNAs at 15 days AFB was observed in the intronic lncRNAs (234.96 RPKM in flowers to 336.53 RPKM at 15 days AFB, *p*-value = 0.0023) and intergenic lncRNAs (525.43 RPKM in flowers and 594.10 RPKM at 15 days AFB, p-value = 0.0399). However, no significant differences were found for the antisense lncRNAs (2484.33 RPKM in flowers and 2046.71 RPKM at 15 days AFB, *p*-value = 0.4484) (Table 2).

Together, these data indicate that the changes in the gene expression level for the specific RNA pol IV and V subunits during fruit development were accompanied by important changes in the expression of lncRNAs, and these changes differed depending on the type of analysed lncRNA.

## 4. Discussion

The role of RNA pol IV and V in plants is still under study and is not fully understood. The olive tree is an important crop and is more complex than the model plant *A. thaliana*. In this work, we searched for the genes encoding the specific subunits of RNA pol IV and V, as well as the RNA pol II from which they have evolved. A comprehensive analysis of the expression profile of these genes was also performed, revealing an increase in mRNA expression for RNA pol IV and V subunits during fruit development. Furthermore, as RNA pol IV and V mediate lncRNA transcription, we analysed lncRNA transcriptome genome-wide and found a positive correlation. In concordance, our data suggest a role for RNA pol IV and V during fruit development through the expression of lncRNAs.

Several genes for the specific RNA pol IV and V subunits, and their RNA pol II counterparts, were identified by a blast-p search with the corresponding *A. thaliana* subunits [7,12,22]. Protein identity with *A. thaliana* homologues varied within the 40–55% range for RNA pol IV and V subunits, except for NRPDE2 with 73% identity (Figure 2). In contrast, the NRPB homologues exhibited notably higher identity levels (79–95%) indicating greater conservation compared to the NRPD and NRPE subunits that have undergone major variation during evolution. RNA pol IV and V have evolved from RNA pol II [4,5,22,62,63], and have apparently evolved more rapidly than the RNA pol II because their *A. thaliana* and *O. europaea* sequences have diverged more.

Several paralogues of the different specific RNA pol II, IV and V subunit genes were found for all the subunits, except for RNA pol IV/V subunit NRPDE2, which had only a single gene (Table 1). This was not the only case for the NRP1, NRP2, NRP4 and NRP7 subunits identified in this work, but also for an additional specific RNA pol V subunit (the previously described NRPE5) which has paralogues for additional common subunits for all the RNA polymerases [28]. The presence of several paralogues is found for many genes in the olive, as is case for the RNA pol subunits shared by the five RNA pols [28]. These results are consistent with the olive cultivar genome that results from two independent whole-genome duplication (WGD) events, in addition to recent partial genome duplications [53,60]. In addition, several paralogues for RNA pol subunits have been identified in other organisms [1,5,22,25,64].

It has been demonstrated that RNA pol IV and V play a role in silencing, plant growth, development, response to environmental changes or plant immunity [4,5,6,7,8,9,10,11,12,22,23,24]. Therefore, we can speculate that the regulation of the gene expression of these RNA pols could be expected in response to some growth conditions. The transcriptomic analysis performed by several RNA-Seq experiments during stress or developing processes using the olive cultivar “Picual” [50,51,52,65] showed that all the genes coding for the specific subunits identified in this work were expressed under these conditions. Similarly, the genes corresponding to the RNA pol II subunits were also expressed. Although no common pattern for the changes in the expression of the RNA pol II, IV or V genes was observed, some cases of regulation by plant tissue were evident. Remarkably, two RNA pol II (NRPB7) genes presented strict organ specificity and were expressed only in flowers (Figure 3 and Figure 4). Furthermore, the response to biotic *V. dahliae* infection [50] or abiotic stresses like cold [51] or root injury [50] showed null or weak changes in the expression profile of most genes.

However, and notably, clear gene regulation occurred for the RNA pol IV and V subunits during fruit development. A consistent and significant overexpression of the RNA pol IV and V genes was observed at the beginning of fruit development, contrasting with the behaviour of the RNA pol II subunits. This observation suggests a possible role of RNA pol IV and V during fruit development in agreement with the role of RNA pol IV and V during plant development and plant growth [12]. According to the role of RNA pol IV and V during the synthesis of ncRNAs, relevant changes in the lncRNAs expression pattern were observed. In line with this, the synthesis of the non-polyA lncRNAs has been demonstrated to be regulated by environmental conditions in *A. thaliana* under abiotic stress [43,44,45]. Indeed, we identified 2303 lncRNAs (non-polyA) transcripts in flowers and fruit at 15 days AFB. The majority of these transcripts were intergenic, while intronic and antisense lncRNAs were less frequent (Figure 5). Relevant changes in expression were found, with 284 lncRNA transcripts expressed only in flowers and 264 only in fruit at 15 days AFB (Figure 6). Furthermore, a tendency toward increased expression was noted in the intronic and intergenic lncRNAs, although this was not observed in the antisense lncRNAs. Considering the role of RNA pol IV and V in the synthesis of the small ncRNAs involved in silencing, we cannot rule out that some of these lncRNAs could be processed to small ncRNAs, which has been reported for other plants [35,40]. Indeed, this fact has been observed for intergenic lncRNAs in *A. thaliana* [40] and, in our case, in 39 intergenic transcripts in olive, which were also identified as putative siRNAs.

In summary, our study identified the genes that code for specific RNA IV and V subunits, and the corresponding ones in RNA pol II, in olive cultivar “Picual”. The expression analysis performed of the different organs/tissues, responses to biotic and abiotic stresses and of the development process revealed that the expression of the RNA pol IV and V genes was induced during the early stages of fruit development. This induction was accompanied by relevant changes in the expression of lncRNAs, particularly an increase in the intergenic and intronic lncRNAs. These changes in the expression of lncRNAs may be important for controlling gene expression during fruit development. In addition, certain intergenic transcripts are susceptible to being processed and becoming siRNAs, which are known to play a role in gene expression control. This reinforces the hypothesis that RNA pol IV and V may contribute to the process of fruit development throughout the synthesis of lncRNAs.

## Figures and Tables

**Table 1:** Coding genes for RNA pol II, IV and V subunits in olive trees (*Olea europaea* L. cv. Picual).

RNA Pol II		RNA Pol IV		RNA Pol V	
NRPB1	*Oleur061Scf2303g05021.1*	NRPD1	*Oleur061Scf8288g09024.1* *Oleur061Scf3115g03008.1* *Oleur061Scf1883g00024.1*	NRPE1	*Oleur061Scf1459g03013.1* *Oleur061Scf0397g00013.1* *Oleur061Scf0194g01004.1*
	*Oleur061Scf0709g00017.1*				
	*Oleur061Scf1475g00008.1*				
	*Oleur061Scf0012g03006.1*				
NRPB2	*Oleur061Scf0169g06007.1* *Oleur061Scf0008g04041.1* *Oleur061Scf3112g05037.1*	NRPDE2	*Oleur061Scf2342g06016.1*		
NRPB4	*Oleur061Scf7473g00034.1* *Oleur061Scf0021g02004.1*	NRPDE4	*Oleur061Scf9139g02012.1*		
			*Oleur061Scf1057g07004.1*		
			*Oleur061Scf2091g00019.1*		
NRPB7	*Oleur061Scf0456g03006.1*	NRPDE7	*Oleur061Scf8086g00007.1* *Oleur061Scf8230g00012.1*		
	*Oleur061Scf1270g16022.1*				
	*Oleur061Scf3490g10013.1*				
	*Oleur061Scf0186g07027.1*				
	*Oleur061Scf0397g02002.1*				
NRPB7-like	*Oleur061Scf4485g00001.1*				
	*Oleur061Scf7934g03011.1*				

Probable pseudogenes with inactivating mutations are shown in red.

**Table 2:** lncRNA expression analysis in flowers and at 15 days AFB.

LncRNA Type	lncRNAs		Average RPKMs		
	Upregulated	Downregulated	Flower	15 Days AFB	*p*-Value
Intergenic	106	237	525.43	594.10	0.0399
Intronic	13	22	234.96	336.53	0.0023
Antisense	24	14	2484.33	2046.71	0.4484
Total	143	273	718.68	733.22	0.8365

## Data Availability

New RNA-Seq of the total RNA data is available at NCBI as BioProject: PRJNA989401.

## Supplementary Materials

The following supporting information can be downloaded at: https://www.mdpi.com/article/10.3390/genes15010001/s1. Figure S1: Expression profile of genes coding for RNA pol II, IV and V subunits in response to root injury; Figure S2: Expression profile of genes coding for RNA pol II, IV and V subunits in response to the fungal infection by *V. dahliae*; Figure S3: Expression profile of genes coding for RNA pol II, IV and V subunits in response to cold stress; Figure S4: Sampling times for RNA-Seq analysis from recently bloomed flowers, developing fruit at 15 days after full bloom and every month from the flowering stage to fully ripe fruit; Table S1: Quality analysis of RNA-seq and reads alignment to the reference transcriptome of olive (*Olea europaea* L. cv. Picual).
