# Supplementary figures and images for: RNA Polymerases IV and V Are Involved in Olive Fruit Development
Source: Genes (Basel). 2023 Dec 19;15(1):1. doi: 10.3390/genes15010001 (PMC10815247; doi:10.3390/genes15010001)

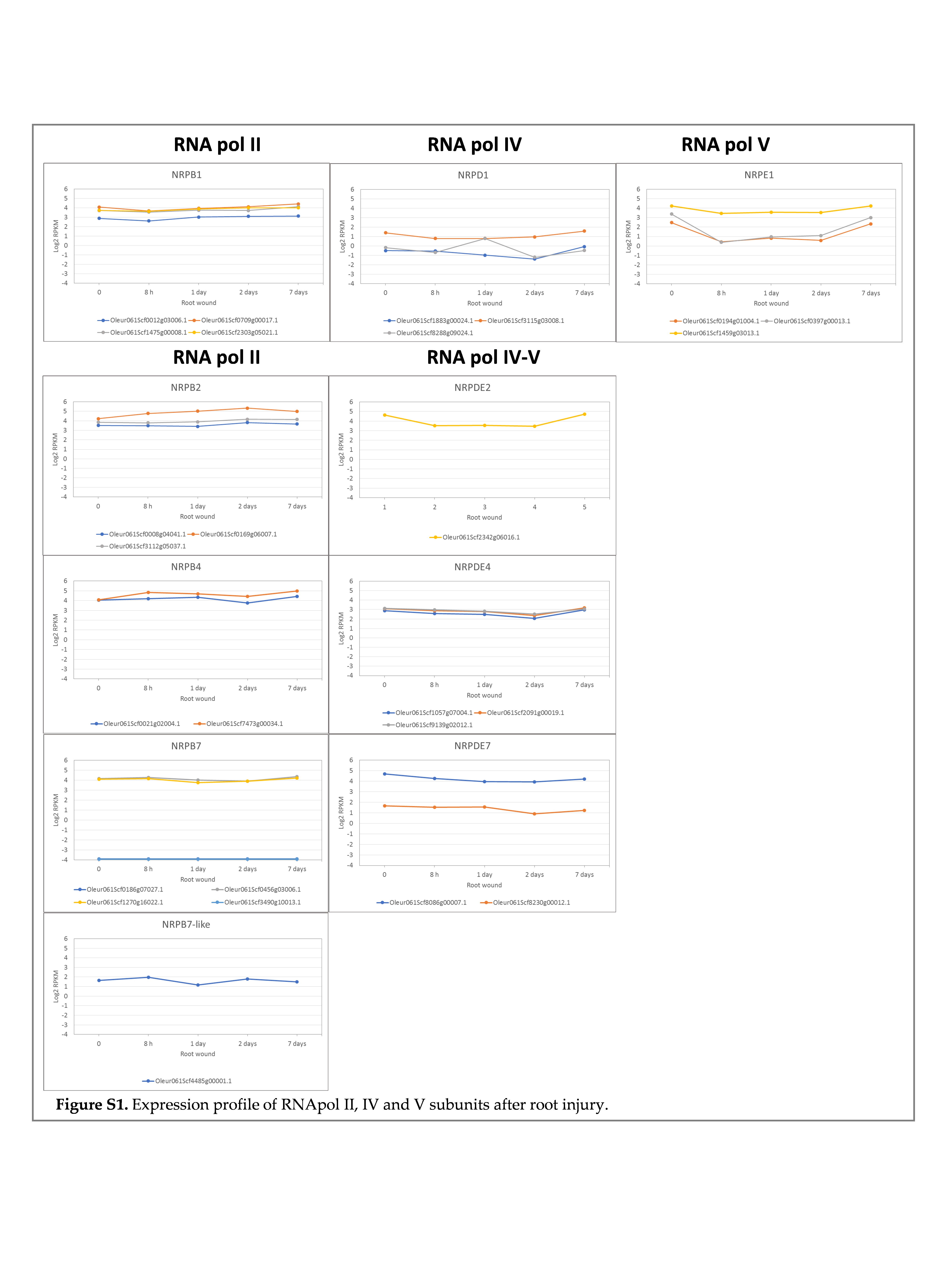

Supplement: Supplementary file 1 [file genes-15-00001-s001.zip › Figure S1.jpg]

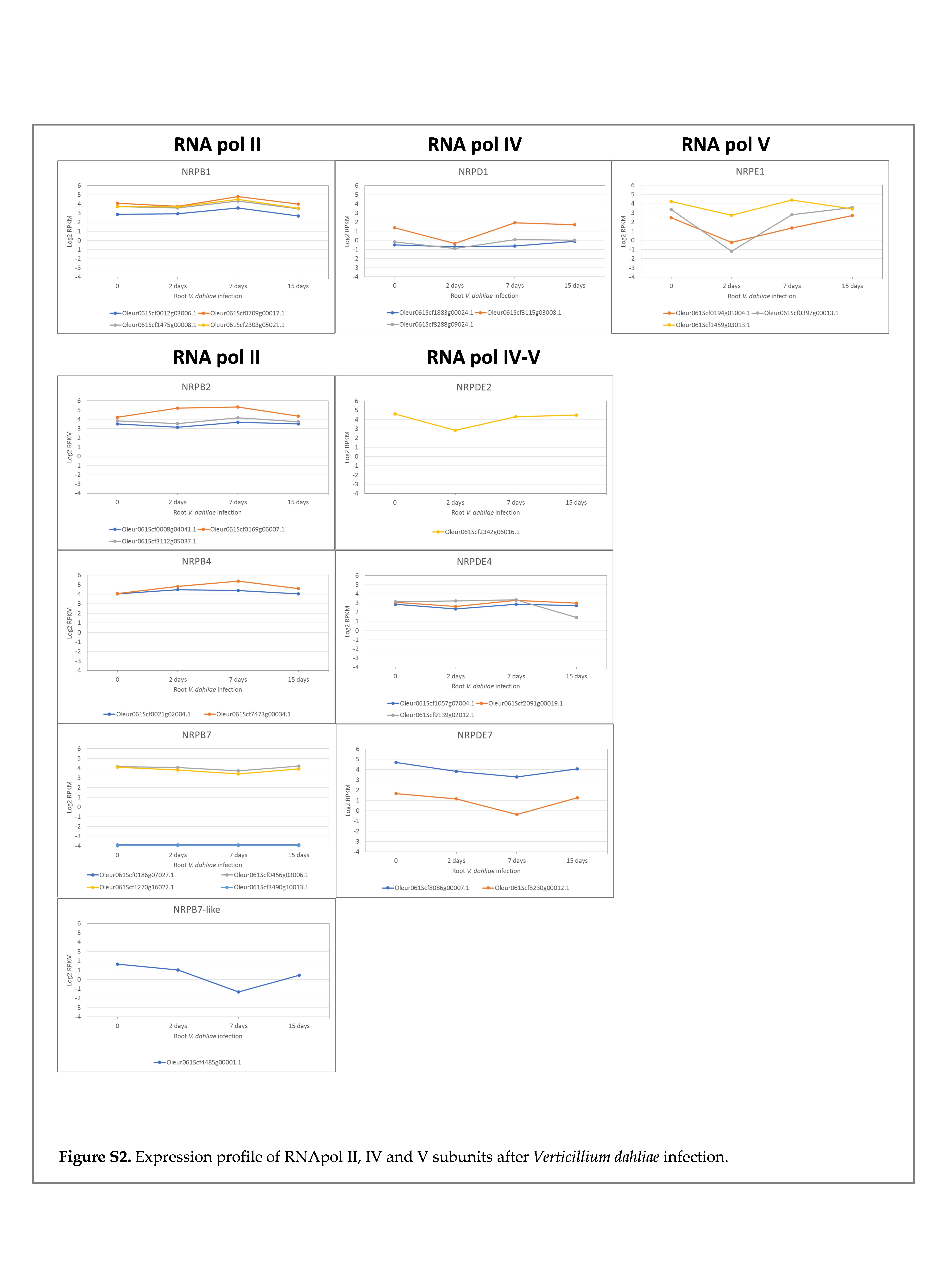

Supplement: Supplementary file 1 [file genes-15-00001-s001.zip › Figure S2.jpg]

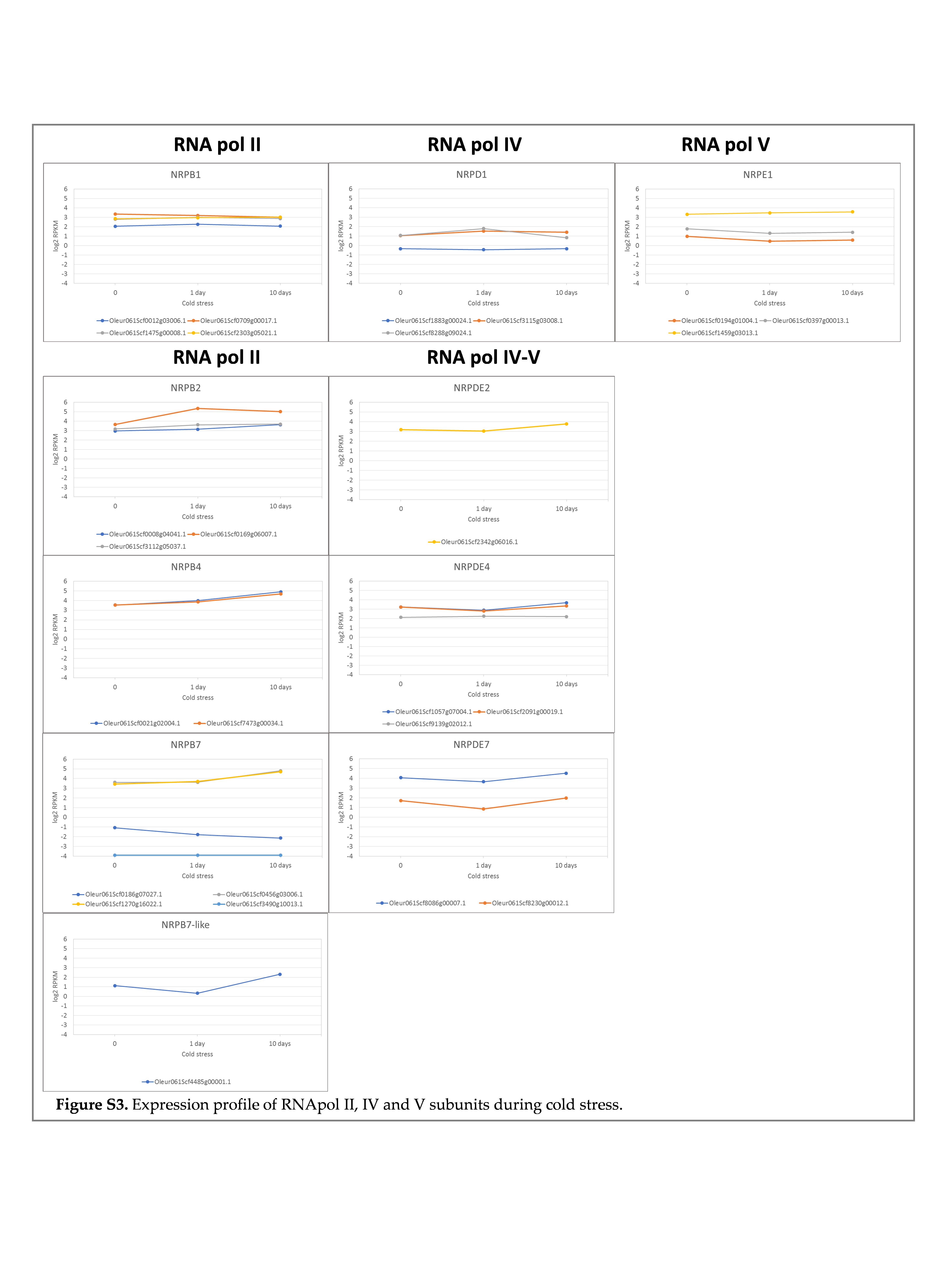

Supplement: Supplementary file 1 [file genes-15-00001-s001.zip › Figure S3.jpg]

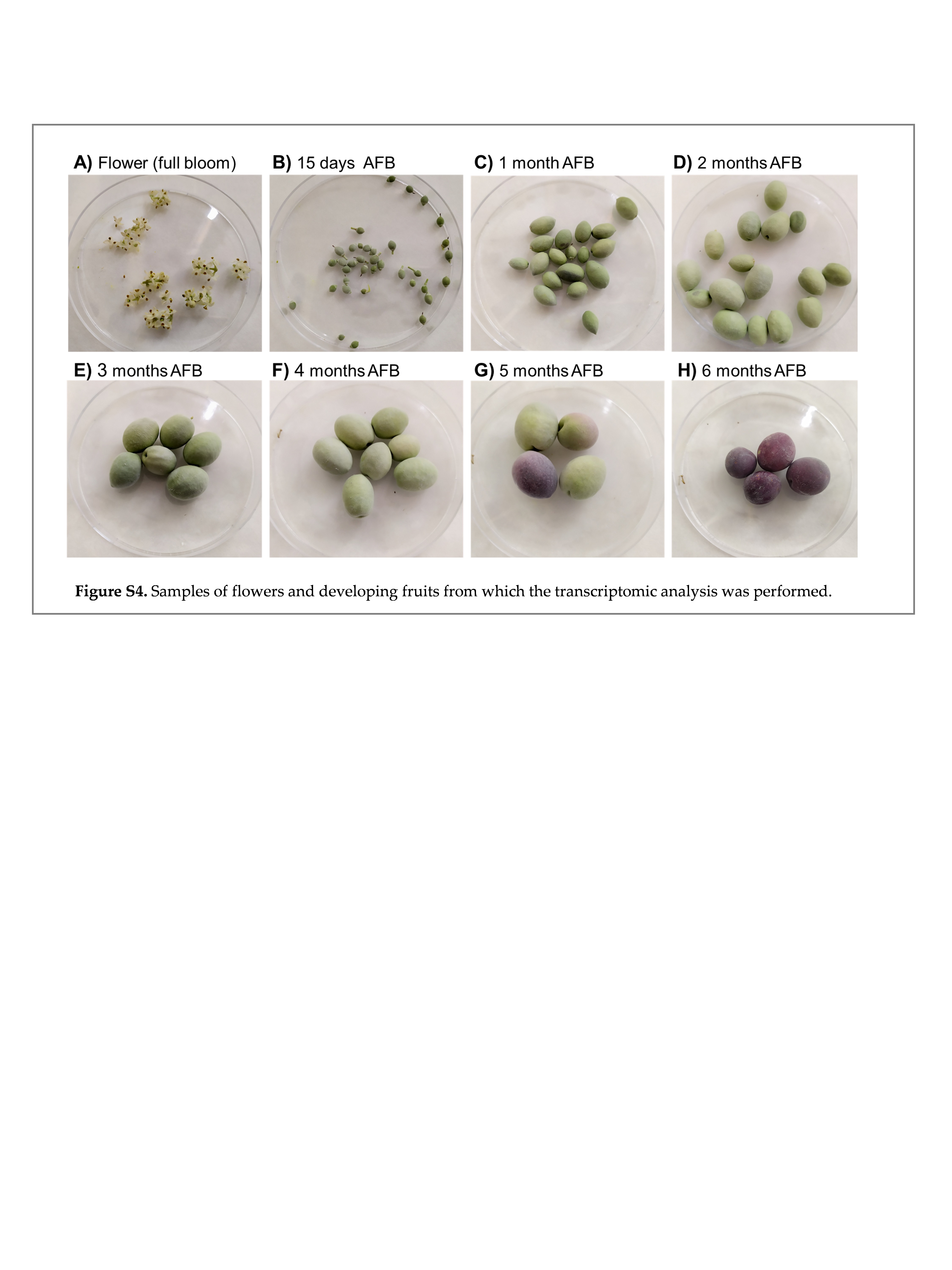

Supplement: Supplementary file 1 [file genes-15-00001-s001.zip › Figure S4.jpg]
